# Estimating the irreversible pressure drop across a stenosis by quantifying turbulence production using 4D Flow MRI

**DOI:** 10.1038/srep46618

**Published:** 2017-04-20

**Authors:** Hojin Ha, Jonas Lantz, Magnus Ziegler, Belen Casas, Matts Karlsson, Petter Dyverfeldt, Tino Ebbers

**Affiliations:** 1 Division of Cardiovascular Medicine, Department of Medical and Health Sciences, Linköping University, Linköping, Sweden; 2 Center for Medical Image Science and Visualization (CMIV), Linköping University, Linköping, Sweden; 3 Division of Applied Thermodynamics and Fluid Mechanics, Department of Management and Engineering (IEI), Linköping University, Linköping, Sweden

## Abstract

The pressure drop across a stenotic vessel is an important parameter in medicine, providing a commonly used and intuitive metric for evaluating the severity of the stenosis. However, non-invasive estimation of the pressure drop under pathological conditions has remained difficult. This study demonstrates a novel method to quantify the irreversible pressure drop across a stenosis using 4D Flow MRI by calculating the total turbulence production of the flow. Simulation MRI acquisitions showed that the energy lost to turbulence production can be accurately quantified with 4D Flow MRI within a range of practical spatial resolutions (1–3 mm; regression slope = 0.91, R^2^ = 0.96). The quantification of the turbulence production was not substantially influenced by the signal-to-noise ratio (SNR), resulting in less than 2% mean bias at SNR > 10. Pressure drop estimation based on turbulence production robustly predicted the irreversible pressure drop, regardless of the stenosis severity and post-stenosis dilatation (regression slope = 0.956, R^2^ = 0.96). *In vitro* validation of the technique in a 75% stenosis channel confirmed that pressure drop prediction based on the turbulence production agreed with the measured pressure drop (regression slope = 1.15, R^2^ = 0.999, Bland-Altman agreement = 0.75 ± 3.93 mmHg).

Stenotic valves and vessels are common pathologies that disrupt the normal flow of blood in the cardiovascular system, and often create turbulent flow and significant pressure losses^1^,^2^. These pressure drops have been an important measure of stenosis severity in the cardiovascular system. For example, the pressure drops occurring across heart valves have been used as thresholds for assessing myocardial load and for determining the necessity of surgical interventions such as aortic valve replacement or transcatheter valve implantation (e.g. aortic and mitral valves)^3^,^4^. The diagnosis and treatment of aortic coarctation are also partially guided by the pressure drop across the lesion^5^. In addition, the mortality of pulmonary hypertension was also associated with the transpulmonary pressure gradient^6^,^7^.

While a direct measurement of the pressure drop using an intravascular pressure catheter is considered the most accurate method, catheterization is not recommended unless non-invasive methods are not available or there appears to be discrepancies between the measured value and the presenting symptoms^3^. As a result, non-invasive Doppler echocardiography is the most commonly used method for estimating the pressure drop. This technique measures the peak velocity in a defined region of interest and estimates the pressure drop using the simplified Bernoulli equation^8^. However, discrepancies between catheter- and Doppler-based pressure measurements are often observed. Based on the assumption of inviscid flow and the ideal energy conversion between kinetic energy and pressure, the simplified Bernoulli equation can overestimate the true irreversible pressure loss by neglecting pressure recovery in the post-stenotic region^9^. In addition, it can underestimate the true pressure difference by not accounting for flow unsteadiness, such as acceleration of the blood flow^10^. Several studies have proposed modified versions of the simplified Bernoulli equation by adding corrective terms. For example, the inclusion of a ratio between the effective aortic orifice area (EOA) and the aortic cross-sectional area to the simplified Bernoulli equation was presented based on the concept of fluid-dynamic energy loss through a sudden expansion, and it has shown the potential to reflect the pressure recovery in the post-stenosis region^11^. Parallel research on the Generalized Bernoulli equation suggested that the accuracy of the pressure drop estimation can be improved by including an acceleration term along a flow streamline upstream and downstream of the stenotic vessel^10^. However, the accuracy of the irreversible pressure drop estimation based on the Bernoulli equation in various pathological conditions remains uncertain^12^,^13^,^14^,^15^.

The advancement of time-resolved, three-directional and three-dimensional phase-contrast magnetic resonance imaging (widely known as 4D Flow MRI) has facilitated the quantification of various hemodynamic features and deeper understanding of complex pathologies^16^,^17^. For example, the quantification of turbulent kinetic energy (TKE) using 4D Flow MRI enabled the examination of chaotic and disturbed flow in a wide range of cardiovascular diseases^1^,^2^,^18^,^19^,^20^. It was also proposed as an alternative index for predicting the irreversible pressure drop across a stenosis by estimating the total amount of TKE, which is correlated to the turbulent energy dissipation and corresponding pressure loss^1^. In spite of the good correlation between TKE and the irreversible pressure drop shown in multiple studies^2^,^9^. TKE based pressure estimation is dependent on both the vessel geometry and stenosis severity which limits its potential^21^. In turbulent flows, energy is drawn from the mean flow to produce turbulence, which in turn is dissipated into heat, based on the dynamic balance between turbulence production and dissipation^22^. TKE reflects the amount of turbulence existing in the flow, but as the dissipation is unknown, it is not sufficient to compute the irreversible pressure drop.

This study demonstrates novel geometry-independent quantification of the irreversible pressure drop across stenoses by quantifying the amount of turbulence production. Building on previous 4D Flow MRI-based turbulence mapping using a six-directional icosahedral (ICOSA6) flow encoding scheme for measuring the complete Reynolds stress tensor^23^,^24^, the feasibility of this technique for the quantification of irreversible pressure loss was investigated in a range of voxel sizes and signal-to-noise ratios (SNR) by simulating 4D Flow MRI based on data from computational fluid dynamics (CFD). The geometry-independency of the estimation of turbulence production and corresponding irreversible pressure drop was investigated using several stenoses. Finally, experimental acquisitions using 4D Flow MRI with ICOSA6 flow encoding were used to demonstrate the assessment of the irreversible pressure drop.

## Results

### Hemodynamic stresses and turbulence production

**Figure 1:**
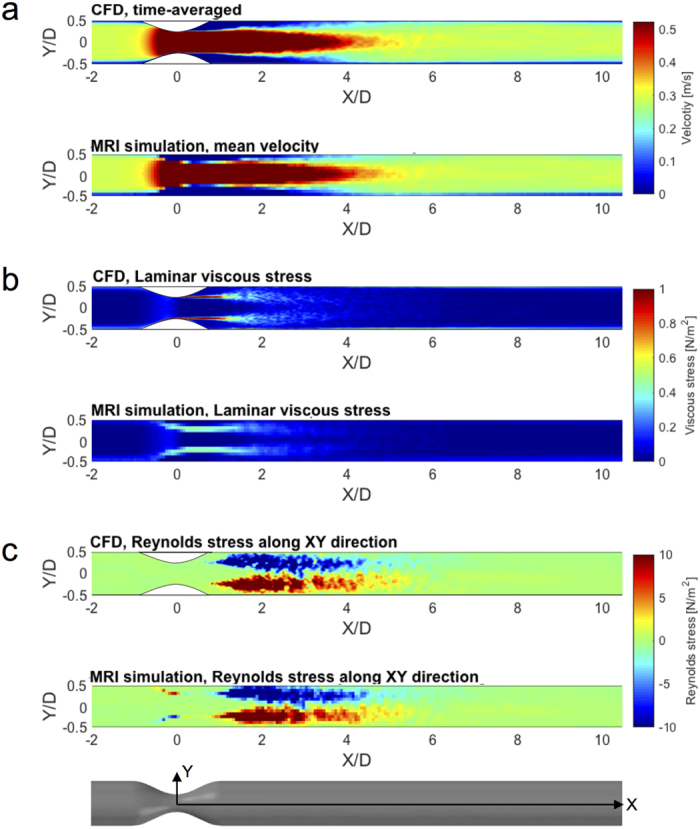
Comparison between CFD and 4D Flow MRI simulation at 75% stenosis with Re = 4000. (**a**) Time-averaged velocity field, (**b**) laminar viscous stress, and (**c**) Reynolds stress component along XY direction. Reynolds stress component along XY direction indicates 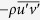 where 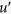 and 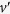 are velocity fluctuations along the X and Y axes, respectively. X and Y are normalized by the upstream diameter (D = 14.6 mm). Principal flow direction is toward the positive X direction. The voxel size for MRI simulation was 1 mm. X and Y-axis are depicted in the bottom panel. Z-axis is the through plane direction.

Time-averaged velocity fields, laminar viscous stresses, and Reynolds stresses were obtained using CFD with large eddy simulation (LES) in a 14.6 mm diameter pipe with a cosine-shaped 75% area-reduction stenosis. The CFD data and corresponding 4D Flow MRI simulation with 1 mm voxels for Reynolds number (Re) = 4000 are compared in Fig. 1. Qualitatively, the simulated 4D Flow MRI and CFD solution agreed for velocity and the primary components of Reynolds stress tensor. The laminar viscous stress in the post-stenosis region, however, was underestimated in the simulated MRI data (Fig. 1b). The maximum value of the laminar viscous stress from simulated MRI was approximately 0.5 N/m^2^, compared to 4.3 N/m^2^ from CFD (Fig. 1b). This occurs mostly due to the limited spatial resolution of 4D Flow MRI compared to the thickness of the shear layer of the jet flow. In contrast, the Reynolds stresses showed good qualitative agreement (Fig. 1c), with the exception of some locally erroneous Reynolds stress at the stenosis apex, due to partial volume effects inherent to MRI.

**Figure 2:**
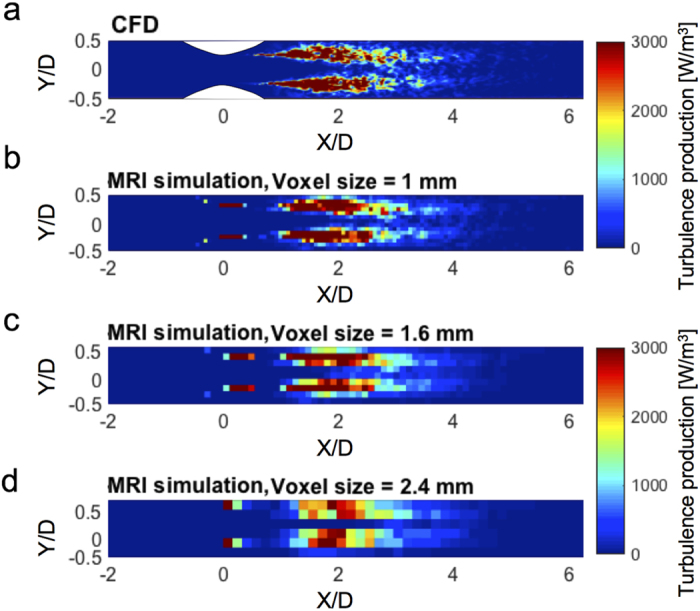
Effects of spatial resolution on turbulence production. (**a**) CFD, (**b**) MRI simulation at 1 mm, (**c**) 1.6 mm, and (**d**) 2.4 mm. Results shown for CFD and simulated MRI at 75% stenosis at Re = 4000. Principal flow direction is toward the positive X direction.

The simulated 4D Flow MRI quantification of turbulence production for spatial resolutions of 1 mm, 1.6 mm, and 2.4 mm are compared with CFD data in Fig. 2. Visual inspection showed that the turbulence production field with higher spatial resolution were closer to the ground-truth CFD (Fig. 2). As the voxel size increased, spatial averaging effects increased and reduced the local maximum value of the turbulence production (Fig. 2; see also Supplementary Figure S1).

**Figure 3:**
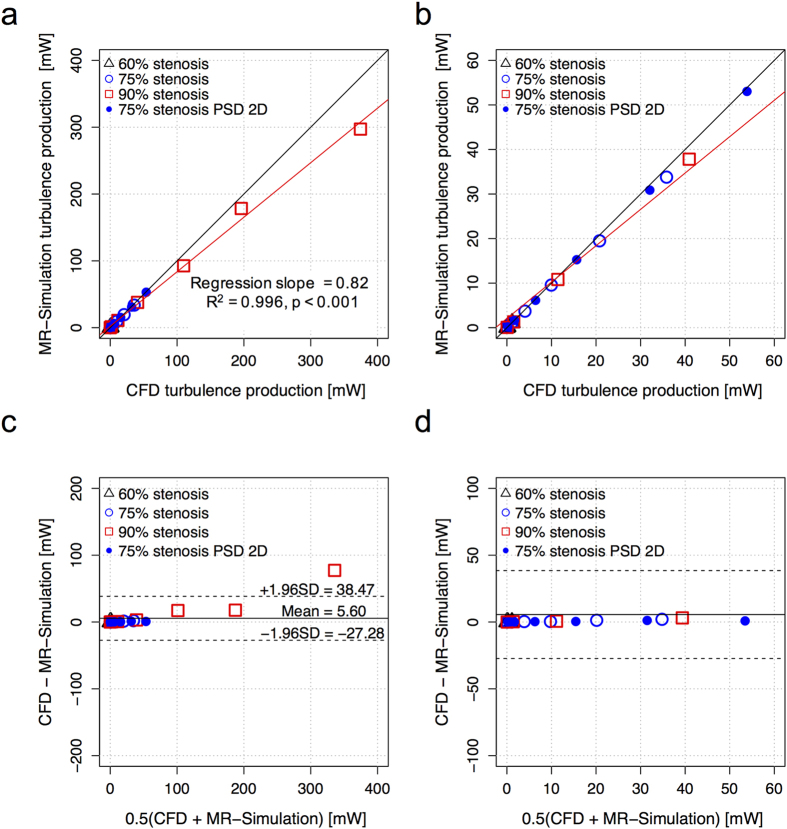
Comparison between total turbulence production from CFD and corresponding 4D Flow MRI simulation. (**a**) Correlation of turbulence production between CFD and MRI simulation, (**b**) enlarged view of plot (**a**,**c**) Bland-Altman plot of total turbulence production, and (**d**) enlarged view of plot (**c**). Each data point is the total sum of the turbulence production. The spatial resolution for MRI simulation was set to 1 mm. Black solid line in (**a** and **b**) indicates unity. Red solid line in (**a** and **b**) indicate the regression lines, which was estimated considering all data points with different stenosis severity and PSD. The solid and dashed lines in (**c** and **d**) represent mean ± 1.96 SD.

Linear regression and Bland-Altman analysis of the total turbulence production in the stenosis at 1 mm spatial resolution showed that the quantification of turbulence production using 4D Flow MRI agreed well with ground-truth CFD solutions, regardless of the stenosis severity or post-stenotic dilatation (PSD) (Fig. 3). The slope of the linear regression for the total turbulence production was 0.82 (p < 0.001, R^2^ = 0.996). The Bland-Altman bias was 5.60 [mW] with 95% limits of agreement spanning from −27.28 to 38.47 [mW].

**Figure 4:**
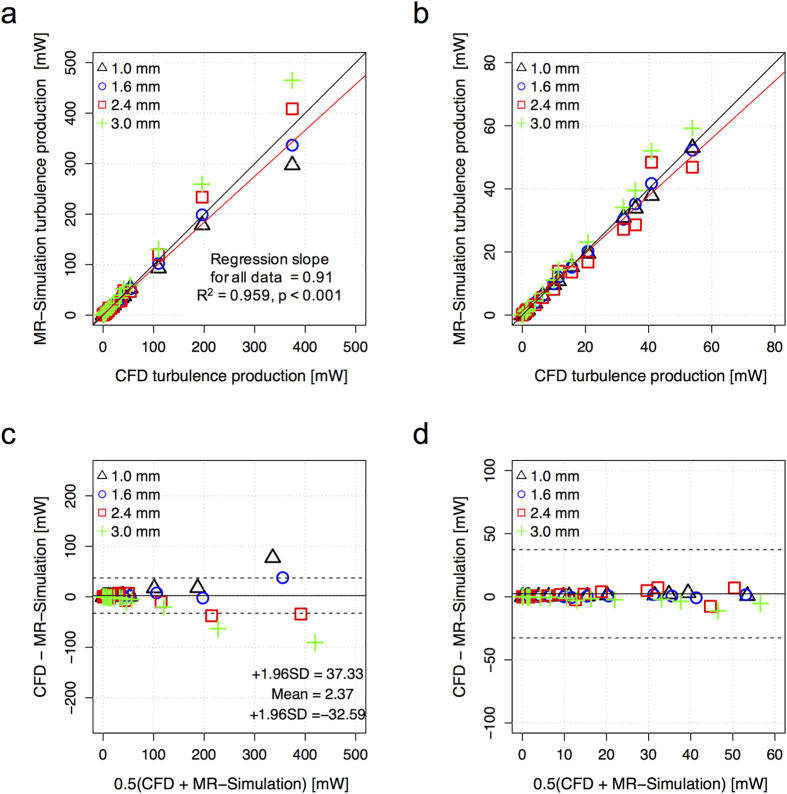
Effects of spatial resolution on the quantification of total turbulence production. (**a**) Correlation of total turbulence production between CFD and MRI simulation, (**b**) enlarged view of plot (**a**,**c**) Bland-Altman plot of total turbulence production, and (**d**) enlarged view of plot (**c**). Black solid line in (**a** and **b**) indicates unity. Red solid line in (**a** and **b**) indicates the regression line, considering all spatial resolutions. The solid and dashed lines in (**c** and **d**) represent mean ± 1.96 SD.

Increased voxel sizes in 4D Flow MRI were found to overestimate the total turbulence production (Fig. 4). The slope of the regression line also increased from 0.82 to 1.26 (0.82, 0.92, 1.11, 1.26 for voxel sizes of 1.0, 1.6, 2.4, and 3.0, respectively). Considering all voxel sizes (1–3 mm), the slope of the regression line for the total turbulence production was 0.91 (p < 0.001) with R^2^ = 0.96. The bias was 2.37 [mW] with limits of agreement: −32.59 and 37.33 [mW]. Most of the discrepancy occurred at total turbulence production >80 mW. For total turbulence production <80 mW the bias was 0.04 with limits of agreement: −4.16 and 4.24 [mW]. Linear regression and Bland-Altman analyses for each voxel size are summarized in Supplementary Table S1.

### Effects of Signal Noise

**Figure 5:**
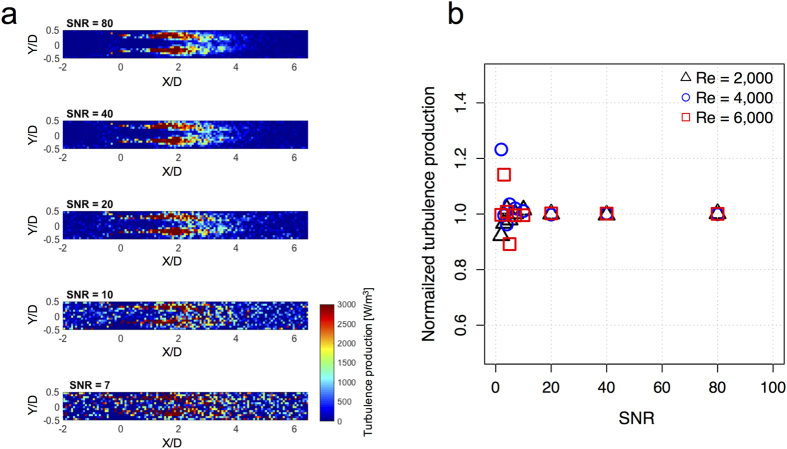
Effects of signal noise on quantification of turbulence production. (**a**) Effects of signal noise on turbulence production powerdensity at 75% stenosis at Re = 4000. X and Y are normalized by the upstream diameter (D = 14.6 mm). Principal flow direction is toward the positive X direction. The voxel size for MRI simulation was 1 mm. (**b**) The effects of signal noise on normalized turbulence production power density. Each data point is the mean of ten repetitions at each SNR. SD were not presented for clarity. SD are described in Supplementary Table S2.

A decrease in the SNR of 4D Flow MRI data leads to increased random noise in the turbulence production field (Fig. 5; see also Supplementary Figure S2). However, when multiple simulations were performed, the mean bias in the total turbulence production was less than 2% with the standard deviation (SD) less than 5% at SNR > 10. At this noise level, the normalized mean ± SD for turbulence production at Re = 2000, 4000, and 6000 were 1.015 ± 0.046, 1.011 ± 0.040, and 0.996 ± 0.028, respectively. The effects of noise on the measurements of turbulence production for Re of 2000–6000 are also summarized in Supplementary Table S2.

### Quantification of irreversible pressure drop across the stenosis

**Figure 6:**
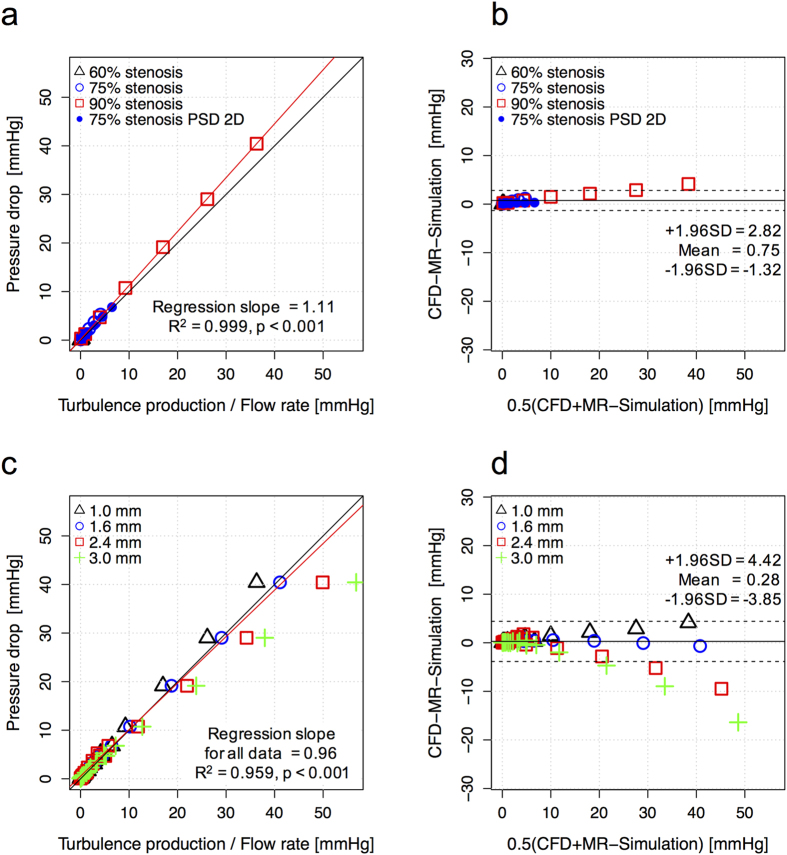
Comparison between the measured irreversible pressure drop across the stenosis and predicted pressure drop based on turbulence production. (**a**) Correlation between irreversible pressure drop between CFD and MRI prediction at 1 mm spatial resolution, (**b**) Bland-Altman plot. (**c**) effects of spatial resolution on the correlation between irreversible pressure drop estimates derived from CFD and MRI, and (**d**) Bland-Altman plot of irreversible pressure drop between CFD and MRI prediction for spatial resolutions 1.0–3.0 mm (**c**). Black and red solid lines in (**a** and **c**) indicate the unity and regression lines, respectively. The solid and dashed lines in (**c** and **d**) represent mean ± 1.96 SD.

4D Flow MRI-based prediction of the irreversible pressure drop across a stenosis based on the total turbulence production agreed with ground-truth CFD solutions at 1 mm spatial resolution, regardless of the stenosis severity or PSD (Fig. 6a,b). The slope of the regression was 1.11 (p < 0.001, R^2^ = 0.999). The Bland-Altman bias was 0.75 [mmHg] with 95% limits of agreement spanning from −1.32 to 2.82 [mmHg].

Overestimation of the total turbulence production in large voxels caused the predicted irreversible pressure drop to be slightly overestimated (Fig. 6c,d). The slope of the regression line was decreased from 1.11 to 0.73 (1.11, 0.99, 0.81, and 0.73 for voxel sizes of 1.0, 1.6, 2.4, and 3.0 mm, respectively). However, considering all voxel sizes tested (1–3 mm), 4D Flow MRI-based prediction of the irreversible pressure drop was strongly correlated to the ground-truth pressure drop. The slope of the regression line for the irreversible pressure drop was 0.96 (p < 0.001) with R^2^ = 0.959. The bias was 0.28 [mmHg] with limits of agreement spanning from −3.85 to 4.42 [mmHg]. Comparison of the irreversible pressure drop assessment for each voxel size is also summarized in Supplementary Table S2.

### *In vitro* validation of irreversible pressure drop assessment

**Figure 7:**
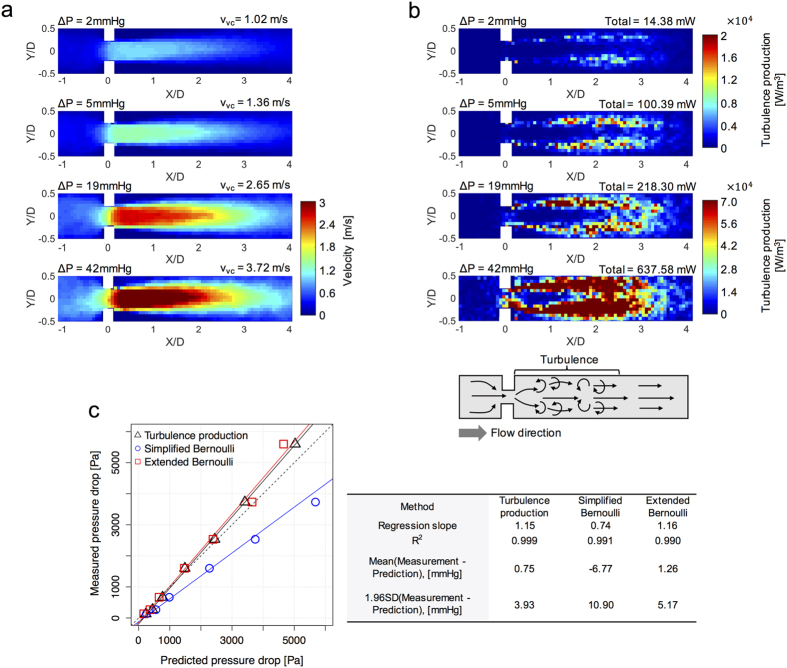
*In vitro* demonstration of 4D Flow MRI based turbulence production quantification for estimating the irreversible pressure drop across the stenotic channel. (**a**) Velocity field with corresponding peak velocity (v_vc_) and measured irreversible pressure drop. (**b**) turbulence production power density and measured irreversible pressure drop. Lower panel of (**b**) depicts a schematic of the 75% constriction channel used in the demonstration and the accompanying turbulence development. (**c**) comparison of pressure drop estimates based on turbulence production, simplified and extended Bernoulli equations. Black, red, and blue solid lines in (**c**) indicate the regression lines between the measured and predicted pressure drop using turbulence production, simplified Bernoulli, and extended Bernoulli methods, respectively. The black dashed line indicates the unity. Right panel of (**c**) describes the correlation and agreement between the measurement and the predicted values.

Velocity fields and turbulence production for the stenosis model were measured with 4D Flow MRI (Fig. 7), and used to predict the corresponding irreversible pressure drop. The measured irreversible pressure drop increased from 1 to 42 [mmHg] as the flow rate increased from 1.3 to 7.6 [L/min] (Table 1). Meanwhile, 4D Flow MRI showed that v_vc_ was increased from 0.69 to 3.71 [m/s], and the total amount of the turbulence production increased from 5.34 [mW] to 637.58 [mW] (Fig. 7a,b).

Comparing the three methods for the pressure drop estimation, the simplified Bernoulli was found to overestimate the irreversible pressure drop (Fig. 7c). The slope of the linear regression was 0.74 (p < 0.001, R^2^ = 0.991). The Bland-Altman bias was −6.77 [mmHg] with 95% limits of agreement spanning from −17.67 to 4.13 [mmHg]. Both the turbulence production method and the extended Bernoulli equation resulted in more accurate prediction than the simplified Bernoulli equation. The slope of the regression for the turbulence production method was 1.15 (p < 0.001, R^2^ = 0.999). The Bland-Altman bias was 0.75 [mmHg] with 95% limits of agreement spanning from −3.18 to 4.68 [mmHg]. The slope of the regression for the extended Bernoulli equation was 1.16 (p < 0.001, R^2^ = 0.990). The Bland-Altman bias was 1.26 [mmHg] with 95% limits of agreement spanning from −3.91 to 6.43 [mmHg].

## Discussion

This study demonstrated the novel application of 4D Flow MRI with ICOSA6 flow encoding for geometry-independent quantification of the irreversible pressure drop across a stenosis. The major findings were that: (1) the turbulence production of the flow can be accurately quantified with 4D Flow MRI within a range of practical spatial resolutions; (2) the quantification of turbulence production was not strongly affected by signal noise; and (3) both simulated 4D Flow MRI and *in vitro* acquisitions demonstrated that the predicted pressure drop across the stenosis based on turbulence production agreed well with the ground-truth pressure drop.

Turbulence in cardiovascular flow is frequently developed as a result of valvular or vascular diseases such as aortic stenosis or aortic coarctation, as well as artificial devices such as prosthetic heart valves, stents, and grafts. An increase of the irreversible pressure drop not only indicates the increased severity of the obstruction, but it also indicates increased cardiac load^1^,^15^. Unfortunately, conventional 2D echocardiography and 2D PC-MRI are both limited in their ability to quantify the complex 3D nature of turbulent blood flow, and require considerable assumption in the estimation of the irreversible pressure drop. In comparison, 4D Flow MRI is capable of quantifying the turbulence production developed through stenoses and the corresponding irreversible pressure drop. Therefore, it appears that 4D Flow MRI can play an important role in the quantification of turbulent blood flow and its effects, both in research and in clinical settings.

While echocardiography is a popular method clinically for assessing the hemodynamic changes associated with cardiovascular disease^3^,^25^, frequent mismatch between the pressure drop estimated using echocardiography and catheter measurements occur because the simplified Bernoulli equation cannot capture the complexity of turbulence in aortic blood flow^12^,^26^,^27^,^28^. Given its relevance for intervention planning, the need to validate methods used for pressure drop assessment has been raised^14^,^29^. As the proposed method is based on the turbulence quantification using 4D Flow MRI, it is an independent measure that can be used to cross-validate the results from echocardiography. Therefore, it could be used to assess the accuracy of the echocardiographic results and increase confidence in clinical decisions drawn from the data.

The theory underlying pressure drop estimation based on turbulence production is different than the theory underlying the simplified Bernoulli equation. The simplified Bernoulli equation assumes amongst other things that the pressure drop across the stenosis is equal to the energy converted from the pressure potential to the kinetic energy of the flow^8^. As it neglects the fluid viscosity, the velocity upstream of the stenosis, and the energy conversion from kinetic energy to pressure potential in the post-stenosis region, the pressure difference indicated by the simplified Bernoulli is the maximum possible pressure drop. This maximum only occurs if there is no pressure recovery at all. To compensate for this inherent overestimation with the simplified Bernoulli equation, an additional corrective term can be added to account for pressure recovery^11^. In contrast, the proposed method quantifies the total turbulence production, which must eventually be dissipated. Therefore, it only quantifies the irreversible net pressure drop. As a result, the proposed method requires neither corrective terms nor geometric descriptions of the stenosis to estimate the irreversible pressure drop. This allows robust irreversible pressure drop estimation even for complex geometries.

*In vitro* tests showed that both the irreversible pressure drop estimations based on the turbulence production and the extended Bernoulli equation were strongly correlated to the measured irreversible pressure drop (Fig. 7). Although both methods accurately predicted the irreversible pressure drop in the simple stenosis model, it is likely they will differ under various clinical conditions. While the pressure drop estimation with the Bernoulli equation assumes that the flow has uniform velocity profile at the stenosis, this assumption is likely to fail for a variety of clinical scenarios. Patients with aortic valve abnormalities, such as those with bicuspid morphology, or severely stenotic valves, frequently have non-uniform eccentric velocity profiles that influence the pressure recovery and the net irreversible pressure drop^30^,^31^,^32^,^33^. For these cases, quantifying the irreversible pressure drop using turbulence production may yield more accurate results as it does not employ the same simplifying assumptions. In addition, the turbulence production based method is not limited to valvular flow, whereas the Bernoulli equation was developed specifically for stenotic valvular flow. Therefore, the quantification of turbulence production can also be used to measure the energy loss and flow efficiency in various non-stenotic flows, such as intracardiac blood flow.

This study found that some overestimation of the total turbulence energy and the corresponding irreversible pressure drop estimation occurred with large voxels. Previously, Binter *et al*. showed that strain-based calculations such as viscous and turbulent loss can be affected by the spatial resolution^34^. The results show that while the volumetric sum of the turbulence production was overestimated for large voxel sizes, the local value of the turbulence production in each voxel was slightly reduced because of spatial averaging effects (Fig. 2). This was mostly driven by the fact that the volumetric sum of the Reynolds stress magnitude was overestimated at the larger voxel size (Supplementary Figure S4). Reynolds stress estimation using 4D Flow MRI assumes that the velocity distribution within a voxel is Gaussian^35^,^36^. However, as the voxel size increases, the velocity distribution is less likely to be Gaussian, which can introduce partial volume-related overestimation of Reynolds stress^2^,^21^. As the estimation of the turbulence production and the corresponding pressure drop prediction is dependent on the voxel size, acquiring multiple datasets with identical spatial resolution would be ideal for comparison. That being said, it is not a strict requirement as both the turbulence production and the corresponding pressure prediction were still strongly correlated for the data ranging from 1 to 3 mm (R^2^ = 0.959, Figs 4 and 6). It is also noteworthy that underestimation of the laminar viscous stress did not result in the underestimation of the turbulence production. Spatial averaging causes the underestimation of the peak local maximum stress, but it does not significantly impact the total volumetric sum of the laminar viscous stress used for the turbulence production estimate.

The quantification of the total turbulence production was not significantly impacted by noise. The influence of SNR on the turbulence production in 4D Flow MRI showed that SNR > 10 resulted in a mean bias of less than 2% with SD less than 5% (Fig. 5, and Supplementary Table S2). Considering that conventional 4D Flow MRI acquisitions have SNR > 30 without the use of a contrast agent^37^, the influence of SNR on the turbulence production in practical 4D Flow MRI measurements will not cause substantial errors.

This study was designed for assessing the pressure drop across large vessels, but the approach is not limited to large vessels. This study investigated a vessel of 14.6 mm with spatial resolutions of 1–3 mm, but the results also correspond to a vessel approximating the aorta (D ≈ 29.2 mm) with spatial resolutions of 2–6 mm or a vessel approximating the carotid artery (D ≈ 7.3 mm) with spatial resolutions of 0.5–1.5 mm, as long as the flow has the same Re number for fluid dynamic similarity^38^. We investigated a wide range of flows (Re: 500–6000) and stenosis severities (60–90%), and therefore we believe that the method presented in this study can be applied in vessels ranging from the aorta to much smaller arteries. The vessel size lower limit is linked to practical issues of MRI acquisition, such as low SNR at high resolution.

One of the limitations of this study is that the effects of pulsatile flow have not been examined experimentally or using CFD. The use of steady flow reduces the complexity for this proof-of-concept study, while allowing the examination of other important parameters, such as the spatial resolution and SNR. Flow pulsatility does not appear likely to affect the performance of the method presented here. Using this method for pulsatile flow measurement requires considering the change in kinetic energy (Eq. 9), taking into account the flow acceleration and convection. A recent study used the similar energy balance equation with the present method for the estimation of peak pressure gradients across the aortic valves^39^. Although they did not calculate the irreversible pressure drop due to the lack of the turbulence energy loss in their calculation, their study shows that the energy balance approach successfully works for the pulsatile flow conditions. Therefore, given that the kinetic energy can be easily obtained from the 4D Flow MRI, further validation of the present method in physiological pulsatile flows would be straightforward.

Another limitation of this study is the limited numbers of stenosis geometries tested. The principal reason behind the use of a simplified stenosis model in this study was that these geometries and corresponding flows have been previously investigated using various methods^21^,^40^. The consistent usage of the same flow dataset is beneficial for understanding the current results based on previous studies, while also eliminating the need for further validation of new CFD data. While this study investigated a simplified stenosis geometry, several stenosis severities (60% to 90%) and two different poststenotic dilatations were examined to take into account of various geometries. As this study uses a simplified geometry, *in-vitro* demonstrations with more complex vascular structures based on *in-vivo* observations can be also performed as previous studies have shown^34^,^41^. As one of the most promising use-cases for the method presented here is the assessment of the irreversible pressure drop across stenotic aortic valves, the performance of this method when examining different aortic valve morphologies such as tricuspid aortic valves, bicuspid aortic valves, and prosthetic aortic valves, is of interest for future study.

This study also lacks of *in-vivo* validations using a catheter-based measurement. Although the invasive catheter-based pressure measurement is not widely preferable, the *in-vivo* validation of this study still can be performed for patients who are planned to have the catheterization^42^,^43^. Also, the performance and clinical applicability of the present method can be examined *in-vivo* by correlating the irreversible pressure drop obtained using the proposed method with severity levels for various cardiovascular diseases in patients as well as the clinical outcome. Lastly, this study investigated the effects of spatial resolution and SNR based on the ideally simulated flow data, and as such, it did not account for acquisition errors such as signal dephasing, background phase-offsets, or aliasing which can affect the quality of the data^16^.

In conclusion, this study developed a novel method for the quantification of the irreversible pressure drop across a stenosis using 4D Flow MRI. Pressure drop assessment based on the turbulence production was successfully demonstrated in both simulated and *in-vitro* acquisitions. Given the wide array of cardiovascular pathologies that create turbulent flow and pressure losses, enabling non-invasive 4D flow MRI to accurately quantify the pressure drop opens up new and clinically useful possibilities.

## Methods

### Computational fluid dynamics

The numerical simulation in this work uses the same stenosis geometry and flow conditions described in Casas *et al*.^21^. Briefly, the stenosis model for CFD was designed to have a cosine-form constriction^44^,^45^. This study analyzed the flows through three different severities of the stenosis (60, 75, and 90% by area), and two different poststenotic dilatations (PSD) with one and two times the upstream diameter (D = 14.6 mm). Detailed information on the geometries and flow conditions used are summarized in Supplementary Table S4.

A steady flow condition was simulated numerically by solving the Navier-Stokes equation in ANSYS CFX 14.5. Anisotropic hexahedral meshes were prepared with ANSYS ICEM 14.5 for computations. The meshes contained between 10–18 million cells, depending on Re^21^. The non-dimensional wall distance y+ was less than unity to ensure resolution sufficient to resolve the near wall turbulent flow^46^,^47^,^48^,^49^.

The velocity field was computed using LES, which has been validated with both laser Doppler velocimetry and direct numerical simulations for the simulation of aortic flow^47^. The simulations employed the Wall-Adapting Local Eddy-viscosity (WALE) subgrid scale model^48^, and numerical schemes were second order accurate. The time step for resolving turbulent flow fluctuations was 50 μs for Re < 4000, and 25 μs for Re > 4000, both of which have been shown to be sufficient for stenotic flow in similar flow regimes^46^,^47^,^48^,^49^. Data sampling started after one second of flow time to avoid initial transient effects.

A fully developed Poiseuille flow and a constant static pressure were given as inlet and outlet boundary conditions, respectively. The inlet and outlet were placed at 4D and 21D upstream and downstream of the stenosis, respectively. A rigid wall obeying the no-slip condition was used for the simulation. The working fluid was water with a constant density of 997 kg/m^3^ and dynamic viscosity of 8.899 × 10^−4^ kg/(m·s).

### Simulated 4D Flow MRI

Simulated 4D Flow MRI measurements based on CFD data are useful for investigating the effects of multiple scan parameters on the results of 4D Flow MRI^21^. In this study, the simulation method was extended to accommodate ICOSA6 flow encoding^21^. Isotropic voxel resolutions between 1 to 3 mm were simulated at 0.2 mm intervals using the raw velocity field obtained using CFD. The velocity at each voxel was obtained through the combination of a Gaussian weighting scheme considering the distance of velocity measurement from the center of the voxel^21^,^50^,^51^:


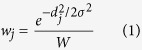


where *w*_*j*_ is the contribution of *j*-th velocity point, *d*_*j*_ is the distance of the *j*-th data point to the center of the voxel, *W* is the total weight within the voxel 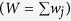, and σ 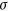
is the variance of the Gaussian function, which is set to ∆*z*/2.35 as described in a previous study^21^.

To simulate the temporal averaging effects of 4D Flow MRI, the velocity at each voxel was calculated by averaging of all velocity data from the transient LES solution using a Gaussian weighting function. More than 400 velocity vectors from the LES solution were averaged for each voxel at the 1 mm spatial resolution, which was considered sufficient^21^.

The velocity signal *s(v*_*i*_) for each direction *i* within the voxel were obtained by estimating the probability density function of velocity within the voxel from a total of 20–30 LES solutions at 10–20 ms intervals. The MRI signal *S*_*i*_(*k*_*v*_) for each velocity distribution *s(v*_*i*_) was calculated by the Fourier-transformation as follows^35^,^36^:


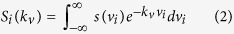


where *k*_*v*_ is flow sensitivity, which is related to the velocity encoding parameter (VENC) as *k*_*v*_ = *π*/*VENC*. The intravoxel variance (IVV) along the direction *i*, 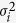, was estimated using the ratio of the reference signal magnitude, *S*_*i*_(*0*), to the magnitude of the velocity encoded signal along the direction *i, S*_*i*_(*k*_*v*_), as follows^35^,^36^:


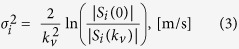


To investigate the effect of noise on the quantification of turbulence production, SNR levels of 2–80 were simulated by adding Gaussian noise to both the real and imaginary signal magnitudes in Eq. [2]. SNR was estimated as the *3σ* of the Gaussian noise distribution per original velocity or signal magnitude. Therefore, the effects of noise on both the mean velocity and IVV were included.

This study extended the previous simulation technique to accommodate 4D Flow MRI with ICOSA6, which employs six directional velocity encodings. The velocity and IVV for each velocity encoding direction were simulated along the encoding directions shown in Supplementary Tables S5 and 6. Least square solutions from the six velocity encodings and IVV were used to extracted the three velocity components (u, v, and w) and full Reynolds stress tensor based on the relationship between the ICOSA6 encoding and the flow components in Cartesian coordinates (Supplementary Tables S5 and 6)^52^.

### Irreversible pressure drop estimation using turbulence production

For the pipe flow system, the rate of work done for the flow is balanced by the net change of kinetic energy (KE) and the rate of energy loss following the energy conservation. The rate of work done by flow through a pipe can be calculated by integrating the rate of work done by pressure over the entire wet surface of the control volume^53^:


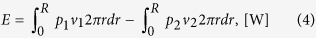


where *E* is the work done by the pressure which is equivalent to the energy dissipation of the flow, *R* is the radius of the pipe, *p*_*1*_, *p*_*2*_ and *v*_*1*_, *v*_*2*_ are the upstream and downstream pressure and velocity, respectively.

Assuming a constant pressure on each cross-sectional area, the energy dissipation and the pressure drop of the flow can be expressed as follows^53^:


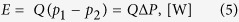


where *Q* is the flow rate, and *∆P* is the pressure drop of the flow.

The energy dissipation in a turbulent flow with large Reynolds number is dominated by the turbulence energy dissipation *ε*^22^. However, its quantification using 4D Flow MRI has not been trivial because the strain rate tensor of the velocity fluctuations cannot be obtained. Instead, the dynamic balance between turbulence dissipation *ε* and turbulent production *P*_*t*_ can be employed as following^22^:


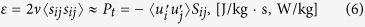


where *v* is the kinematic viscosity, *s*_*ij*_ and *S*_*ij*_ are the strain rate tensor of velocity fluctuation and mean velocity field, respectively. 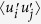 is the Reynolds stress component, and the product of *S*_*ij*_ and the fluid viscosity is the laminar viscous stress. The subscripts *i* and *j* represent the perpendicular directions x, y and z. Therefore, the energy dissipation per unit mass can be estimated from the product of the Reynolds stress and the strain rate tensor of the mean velocity field, which can be measured with ICOSA6 flow encoding. The balance between *ε* and *P*_*t*_ is approximately equal on the local level, but it will be exactly the same after integrating over the entire turbulent region. In this study, *P*_*t*_ was regarded as the dominant source of energy loss because the turbulent fluctuating strain is rate is much larger than mean strain rate 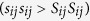
22. *P*_*t*_ in the stenotic flow is assumed to always be positive as discussed elsewhere^54^,^55^. Therefore, intermittent negative *P*_*t*_ values were set to zero to filter possible measurement errors arising from partial volume effects and higher order motion.

The product of the fluid’s density and *P*_*t*_ is the power density of turbulence production with units J/(m^3^·s) or W/m^3^, and its volumetric integration results in the total turbulence production within the region of interest:


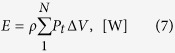


where ρ is the density of the fluid, N is the total number of voxels within the region of interest and *ΔV* is the volume of each voxel. Given that KE is defined as 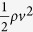, where v is the fluid velocity, the total energy balance in the flow can be described as follows^38^,^55^:


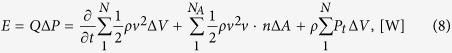


where the first term describes the rate of increase of KE within the control volume, the next describes convection of KE, and the final term is the total turbulence production. A and N_A_ indicate the surface area of the vessel and the total number of surface elements, respectively. n is the unit vector normal to the surface. Given that the net contribution of KE is zero for steady pipe flow, the pressure drop due to the turbulence energy dissipation at steady flow can be simplified as follow:


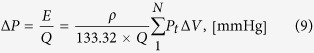


where 133.32 is the conversion factor between from Pascal to mmHg.

### Bernoulli equation for the pressure drop estimation

The simplified Bernoulli equation has been widely used in medicine, and takes the form:


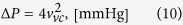


where *v*_*vc*_ is the velocity at velocity at the vena contracta, often calculated as the maximum velocity. More recently, the extended Bernoulli equation has been derived to account for the pressure recovery in the post-stenosis region^11^:


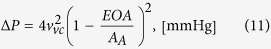


where EOA is the effective orifice area, and A_A_ is the cross-sectional area of the aorta. EOA can be obtained using the continuity equation by assuming a flat velocity profile, Q_p_ = EOAv_vc_, where Q_p_ is the flow rate through the vena contracta, which is estimated proximal to the stenosis^56^.

### *In vitro* validation of irreversible pressure drop assessment

Experimental measurement with 4D Flow MRI was perform to confirm the feasibility of the irreversible pressure drop assessment based on the turbulence production quantification. The stenotic phantom for the experiment was a sudden contraction/expansion model with 75% reduction in area (50% in diameter, see Fig. 7 and Supplementary Figure S4). The upstream diameter without any constriction was 14.6 mm. To fully develop Poiseuille flow upstream of the stenosis, a 1 m straight section was used. Downstream from the constriction was also straight. The pressure was measured at 1D and 10D both upstream and downstream of the stenosis using the blood pressure monitoring system (Xtrans, Codan, Lensahn, Germany).

The working fluid was a blood analog composed of 26.4:73.6 glycerol/water mixture (by mass). The density and dynamic viscosity of the working fluid were 1056.2 kg/m^3^ and 2.04 × 10^–3^ kg·m^−1^·s^−1^, respectively. The working fluid was circulated through the flow circuit system at a constant flow rate using a gear pump (ECO Gearchem G6, Pulsafeeder, NY). The flow rate was controlled from 1.31 to 7.61 L/min, which corresponds to Re of 989 to 5724. Re is expressed as Re = QD/(*ν*A), where Q is the flow rate, D is the diameter of the channel, *ν* is the kinematic viscosity, and A is the cross-sectional area of the channel. The temperature of the fluid during the experiment was 22 °C.

4D Flow MRI measurements were performed using a clinical 1.5T MRI scanner (1.5T Philips Achieva, Philips Medical Systems, Best, The Netherlands). A conventional gradient-echo sequence with asymmetric four-point flow encoding was modified to have six-directional icosahedral flow encoding, with one flow-compensated reference encoding. The velocity encoding parameter (VENC) was varied from 100 cm/s to 589 cm/s for the velocity measurements, and from 32 cm/s to 160 cm/s for the turbulence measurements, depending on the flow rate. Echo time and repetition time were 1.93–3.46 ms and 4.17–5.70 ms, respectively. The flip angle was 10°. The field of view was 336 mm × 336 mm × 180 mm with a 1 mm isotropic voxel size. Partial echo acquisition with a factor of 0.725 along the frequency-encoding directions was used, and the reconstructed with the zero-filling.

Three separate scans for each flow condition were measured. The first and second scans were performed with a different VENC to optimize the sensitivity of the velocity and the turbulence measurement, respectively. The third scan was performed with the same scan parameters as the velocity measurement, but with the pump turned off. The velocity field obtained from the third scan was used to correct the velocity offsets caused by background phase errors^57^. At the cost of increased scan time due to the increased number of flow encodings, 4D Flow MRI with ICOSA6 could estimate three velocity components and Reynolds stress as well as the full turbulence tensor. A schematic detailing the procedure for data analysis is also described in Supplementary Figure S4.

### Statistical Analysis

Volumetric integration of turbulence production and the corresponding irreversible pressure drop prediction from 4D Flow MRI were compared against ground-truth CFD and experimental data. Linear regression was analyzed to assess the relationship between the ground-truth and the prediction from 4D Flow MRI. Slope and coefficient of determination of the regression line (R^2^) were calculated using RStudio (RStudio, Inc., Boston, MA). Bland-Altman analysis was also used to evaluate the agreement between the ground-truth and predicted value.

## Additional Information

**How to cite this article:** Ha, H. *et al*. Estimating the irreversible pressure drop across a stenosis by quantifying turbulence production using 4D Flow MRI. *Sci. Rep.* **7**, 46618; doi: 10.1038/srep46618 (2017).

**Publisher's note:** Springer Nature remains neutral with regard to jurisdictional claims in published maps and institutional affiliations.

## Supplementary Material

Supplementary Information

## Figures and Tables

**Table 1:** Summary of *in vitro* validation of 4D Flow MRI for pressure drop assessment.

Q [L/min]	Re	v_vc_ [m/s]	EOA [m^2^]	A_A_ [m^2^]	Total turbulence production [mW]	ΔP_m_ [mmHg]	ΔP_prod_ [mmHg]	ΔP_SB_ [mmHg]	ΔP_EB_ [mmHg]
1.31	989	0.69			5.14	1	1.8	1.9	1.2
1.94	1456	1.02			14.38	2	3.3	4.2	2.7
2.50	1878	1.36			32.02	5	5.8	7.4	4.9
4.06	3053	2.07	3.19 × 10^−5^	1.67 × 10^−4^	100.39	12	11.1	17.1	11.0
5.36	4034	2.65			218.30	19	18.3	28.1	17.9
6.53	4912	3.26			371.39	28	25.6	42.6	27.3
7.61	5724	3.71			637.58	42	37.7	55.1	34.9

*Q*, flow rate; Re, Reynolds number; v_vc_, velocity at the vena contracta; EOA, effective orifice area; A_A_, cross-sectional area of non-stenotic channel; ΔP_m_, measured pressure drop; ΔP_prod_, predicted pressure drop from turbulence production; ΔP_SB_, predicted pressure drop from the simplified Bernoulli equation; ΔP_EB_, predicted pressure drop from the extended Bernoulli equation.
