# Supplementary material for: Estimating the irreversible pressure drop across a stenosis by quantifying turbulence production using 4D Flow MRI
Source: Sci Rep. 2017 Apr 20;7:46618. doi: 10.1038/srep46618 (PMC5397859; doi:10.1038/srep46618)
Supplement: Supplementary Information [file srep46618-s1.pdf]

Estimating the irreversible pressure drop across a stenosis by quantifying turbulence production using 4D Flow MRI

Hojin Ha[1,2], Jonas Lantz[1,2], Magnus Ziegler[1,2], Belen Casas[1,2], Matts Karlsson[2,3], Petter Dyverfeldt[1,2], Tino Ebbers[1,2]

[1]Division of Cardiovascular Medicine, Department of Medical and Health Sciences, Linköping University, Linköping, Sweden.
[2]Center for Medical Image Science and Visualization (CMIV), Linköping University, Linköping, Sweden.
[3]Division of Applied Thermodynamics and Fluid Mechanics, Department of Management and Engineering (IEI), Linköping University, Linköping, Sweden.

Corresponding Author: Hojin Ha

Phone: +46-762693607

E-mail: hojin.ha@liu.se

Running title: Assessment of irreversible pressure drop using 4D flow MRI.

Number of words: 5541 words

Number of items: 7 Figures, 1 Table

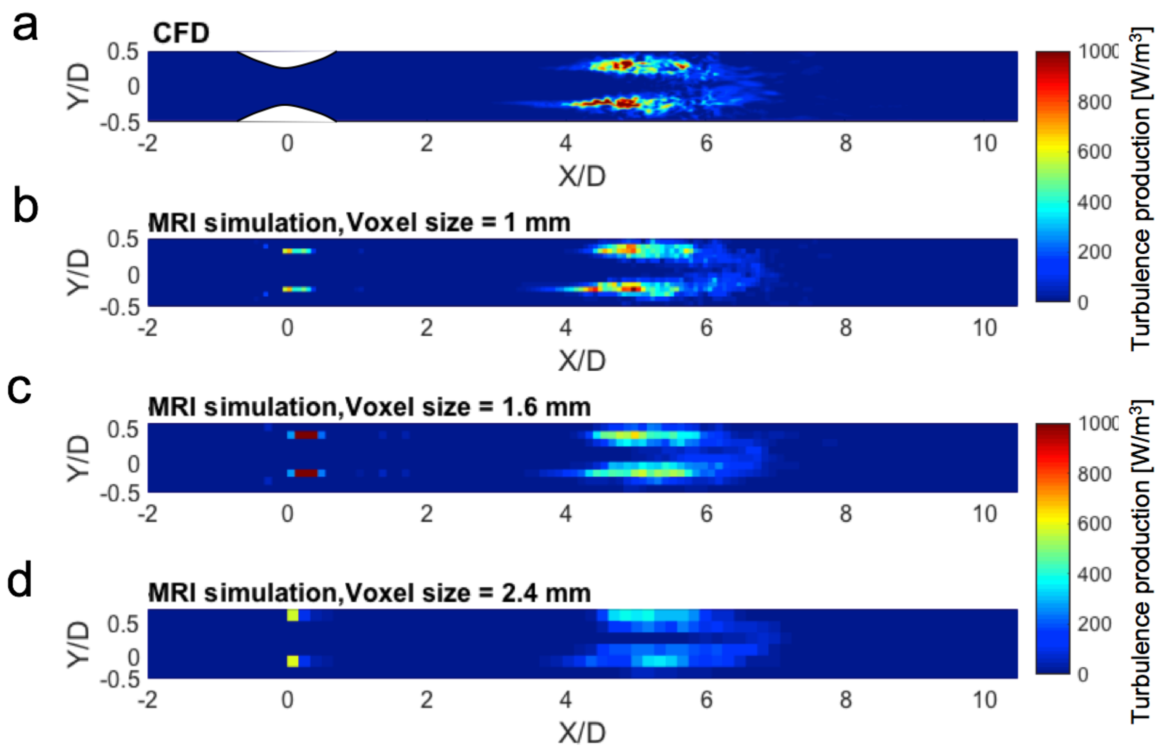

Figure S1. Effect of voxel resolution on turbulence production energy density. (a) CFD, (b) MRI simulation with 1 mm, (c) 1.6 mm and (d) 2.4 mm. Results shows CFD and MRI simulation at 75% stenosis with Re = 2000. Principal flow direction is toward the positive X direction.

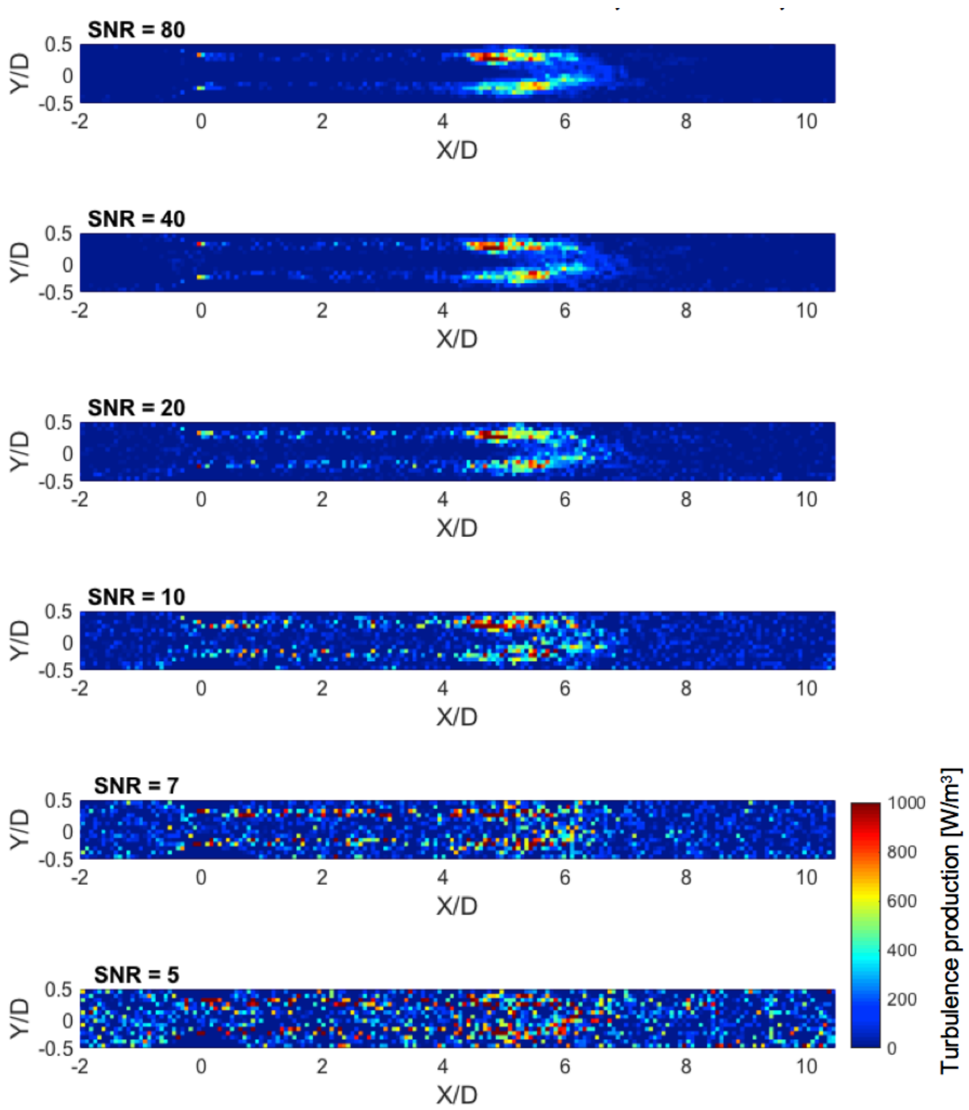

Figure S2. Effect of SNR on turbulence production energy density at 75% stenosis at Re = 2000. X and Y are normalized by the upstream diameter (D = 14.6 mm). Principal flow direction is toward the positive X direction. The voxel size for MRI simulation was set to 1 mm.

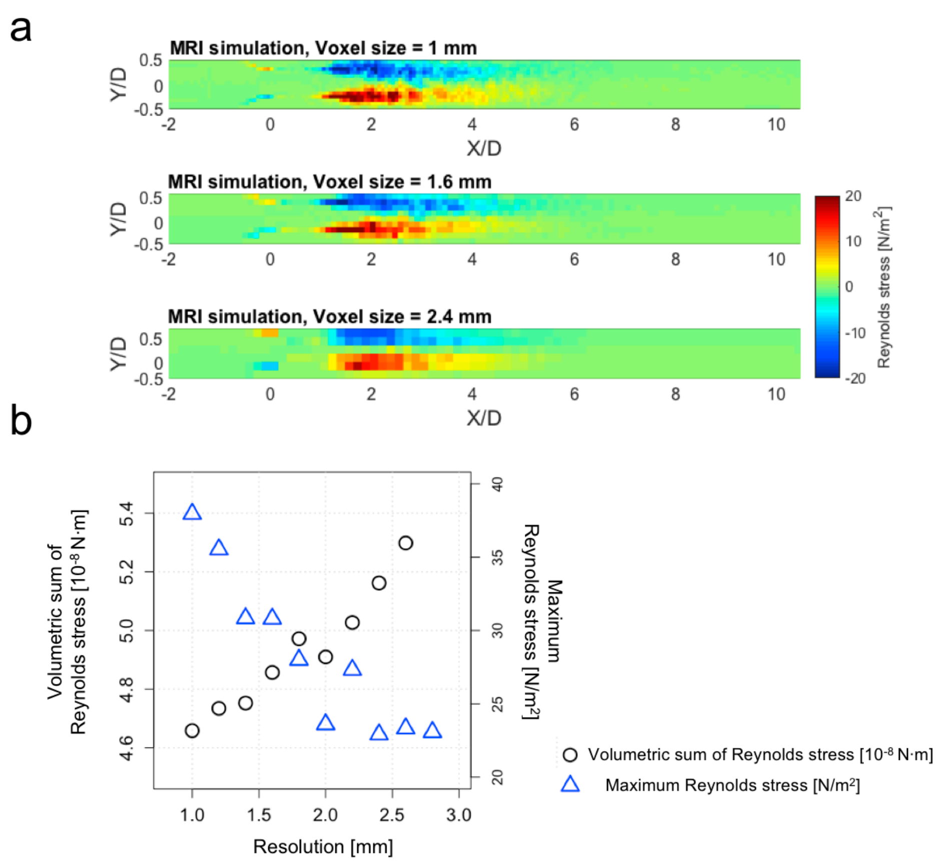

Figure S4. Effect of voxel resolution on the Reynolds stress. (A) 4D flow MRI simulation of Reynolds stress at 1 mm, 1.6 mm and 2.4 mm. Results shows MRI simulation at 75% stenosis with Re = 4000. (B) volumetric sum of Reynolds stress magnitude and maximum Reynolds stress.

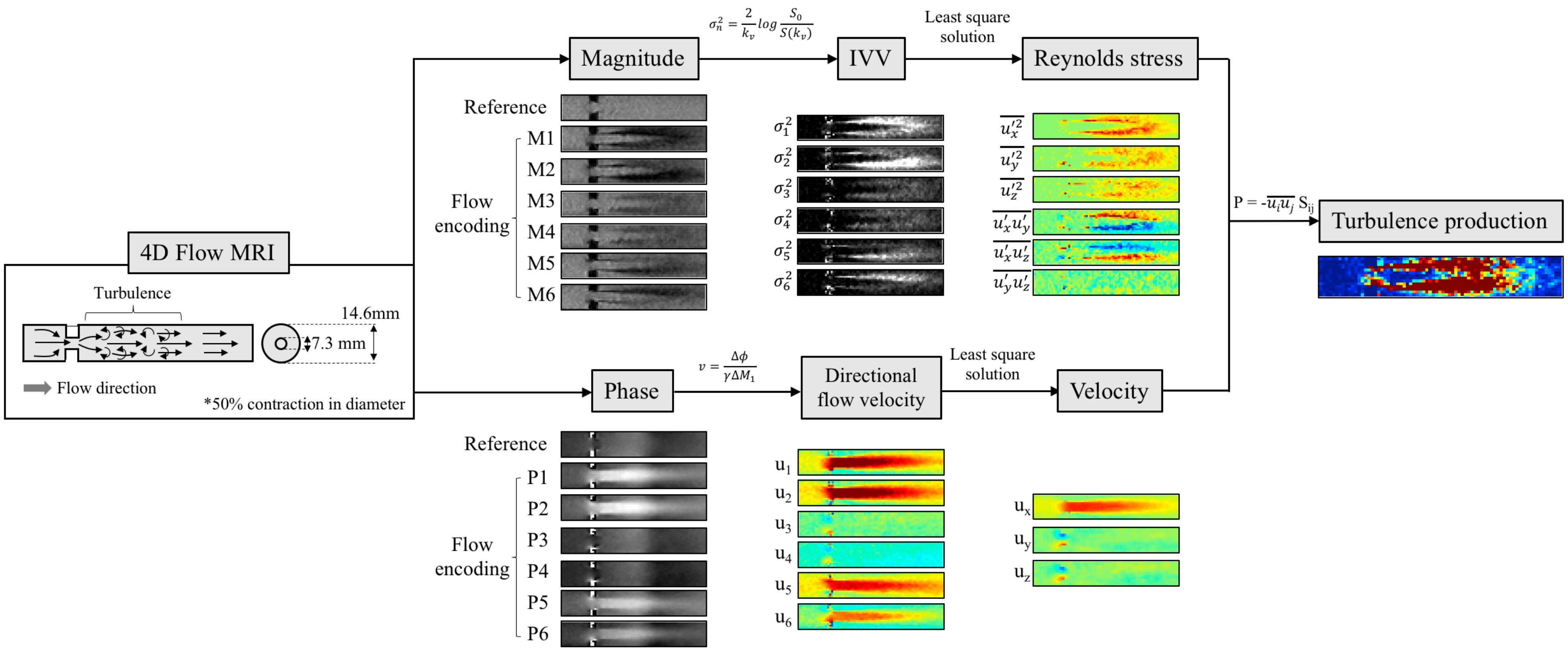

Figure S4. Schematic procedure of turbulence production quantification. The inset in 4D flow MRI shows the geometry of 50% constriction model used in the present study for the experimental demonstration. IVV indicates the intravoxel variance, which is the square of the intravoxel standard deviation.

Table S1. Effect of voxel resolution on turbulence production quantification

Resolution [mm]	Slope	Standard error of slope	Intercept	Standard error of intercept	R^2	P-value	mean(CFD-MRI) [mW]	1.96SD(CFD-MRI) [mW]
1.0	0.82	0.01	2.07	1.17	0.996	<0.001	5.60	32.88
1.2	0.91	0.01	1.46	1.18	0.996	<0.001	2.39	18.42
1.4	0.86	0.01	1.19	0.97	0.997	<0.001	4.83	25.95
1.6	0.92	0.01	1.15	1.06	0.997	<0.001	2.15	16.02
1.8	0.81	0.01	2.65	1.20	0.995	<0.001	5.27	33.92
2.0	0.98	0.01	0.50	0.90	0.998	<0.001	0.49	8.32
2.2	1.03	0.01	0.29	0.94	0.998	<0.001	-1.57	9.19
2.4	1.11	0.01	-1.82	1.38	0.997	<0.001	-2.86	22.23
2.6	0.65	0.02	4.55	1.65	0.986	<0.001	10.24	62.19
2.8	0.70	0.01	3.63	1.31	0.992	<0.001	8.90	52.53
3.0	1.26	0.01	-1.34	1.03	0.999	<0.001	-9.34	44.63
All data	0.91	0.01	1.30	1.16	0.959	<0.001	2.37	34.96

Table S2. Effect of SNR on turbulence production quantification

Reynolds number	SNR	Turbulence production [mW]		Normalized turbulence production	
		Mean	SD	Mean	SD
2000	Inf	1.027	0.000	1.000	0.000
2000	80	1.029	0.004	1.002	0.004
2000	40	1.024	0.006	0.997	0.006
2000	20	1.028	0.021	1.000	0.020
2000	10	1.043	0.048	1.015	0.046
2000	7	1.028	0.081	1.001	0.078
2000	5	1.006	0.128	0.979	0.125
2000	4	1.046	0.138	1.018	0.134
2000	3	0.993	0.294	0.967	0.286
2000	2	0.948	0.659	0.923	0.642
3000	Inf	3.709	0.000	1.000	0.000
3000	80	3.711	0.011	1.001	0.003
3000	40	3.720	0.025	1.003	0.007
3000	20	3.702	0.068	0.998	0.018
3000	10	3.705	0.131	0.999	0.035
3000	7	3.714	0.193	1.001	0.052
3000	5	3.789	0.372	1.022	0.100
3000	4	3.626	0.753	0.978	0.203
3000	3	4.082	0.979	1.101	0.264
3000	2	3.405	2.027	0.918	0.547
4000	Inf	9.567	0.000	1.000	0.000
4000	80	9.559	0.045	0.999	0.005
4000	40	9.583	0.036	1.002	0.004
4000	20	9.547	0.133	0.998	0.014
4000	10	9.669	0.381	1.011	0.040
4000	7	9.763	0.581	1.020	0.061
4000	5	9.904	0.970	1.035	0.101
4000	4	9.206	1.534	0.962	0.160
4000	3	9.523	2.459	0.995	0.257
4000	2	11.790	4.479	1.232	0.468
5000	Inf	19.495	0.000	1.000	0.000
5000	80	19.494	0.059	1.000	0.003
5000	40	19.454	0.104	0.998	0.005
5000	20	19.539	0.193	1.002	0.010
5000	10	19.168	0.478	0.983	0.025
5000	7	19.636	0.648	1.007	0.033
5000	5	20.162	1.444	1.034	0.074
5000	4	19.326	2.479	0.991	0.127
5000	3	20.498	3.037	1.051	0.156
5000	2	15.382	12.931	0.789	0.663
6000	Inf	33.788	0.000	1.000	0.000
6000	80	33.810	0.116	1.001	0.003
6000	40	33.844	0.197	1.002	0.006
6000	20	33.866	0.667	1.002	0.020
6000	10	33.650	0.954	0.996	0.028
6000	7	33.629	1.953	0.995	0.058
6000	5	30.135	2.894	0.892	0.086
6000	4	34.070	4.534	1.008	0.134
6000	3	38.572	9.390	1.142	0.278
6000	2	33.693	14.817	0.997	0.439

Table S3. Effect of voxel resolution on irreversible pressure drop estimation based on turbulence production quantification

Resolution [mm]	Slope	Standard error of slope	Intercept	Standard error of intercept	R^2	P-value	mean(CFD-MRI) [mmHg]	1.96SD(CFD-MRI) [mmHg]
1.0	1.11	0.01	0.15	0.06	0.999	<0.001	0.75	2.07
1.2	1.00	0.01	0.28	0.06	0.999	<0.001	0.27	0.49
1.4	1.06	0.01	0.31	0.09	0.999	<0.001	0.66	1.41
1.6	0.99	0.01	0.32	0.07	0.999	<0.001	0.24	0.63
1.8	1.12	0.01	0.04	0.07	0.999	<0.001	0.70	2.25
2.0	0.93	0.01	0.43	0.13	0.997	<0.001	0.03	1.77
2.2	0.88	0.01	0.46	0.11	0.998	<0.001	-0.31	2.85
2.4	0.81	0.01	0.74	0.18	0.995	<0.001	-0.50	4.84
2.6	1.41	0.03	-0.48	0.26	0.991	<0.001	1.46	6.18
2.8	1.30	0.02	-0.24	0.16	0.997	<0.001	1.25	4.86
3.0	0.73	0.01	0.66	0.17	0.996	<0.001	-1.43	7.77
All data	0.96	0.01	0.53	0.15	0.959	<0.001	0.28	4.14

Table S4. Geometry and flow conditions for the numerical simulation and 4D flow MRI simulation

Severity[a] (%)	PSD[b]	Reynolds number
60	-	1000
60	-	2000
60	-	3000
75	-	1000
75	-	2000
75	-	3000
75	-	4000
75	-	5000
75	-	6000
90	-	500
90	-	1000
90	-	2000
90	-	3000
90	-	4000
90	-	5000
90	-	6000
75	2D	1000
75	2D	2000
75	2D	3000
75	2D	4000
75	2D	5000
75	2D	6000

[a]Severity is the percentage of area reduction at the stenosis apex.
[b]Post-stenotic dilatation (PSD), defined as a ratio between a diameter at the post-stenosis and upstream diameter.

Table S5. Flow encoding scheme with ICOSA6 sequence

Number of encoding	Conventional 4D flow MRI	ICOSA encoding
0	0	0
1	$\Delta M_1(x)$	$\Delta M_1(\cos\theta^{b}\cdot x + \sin\theta\cdot y)$
2	$\Delta M_1(y)$	$\Delta M_1(\cos\theta\cdot x - \sin\theta\cdot y)$
3	$\Delta M_1(z)$	$\Delta M_1(\cos\theta\cdot y + \sin\theta\cdot z)$
4	NA[a]	$\Delta M_1(\cos\theta\cdot x - \sin\theta\cdot y)$
5	NA	$\Delta M_1(\sin\theta\cdot x + \cos\theta\cdot z)$
6	NA	$\Delta M_1(\sin\theta\cdot x - \cos\theta\cdot z)$

[a]NA; not-applicable, [b] θ for the present study is about 31.17º, which corresponds to cosθ = 0.8507 and sinθ = 0.5257

Table S8. Velocity and intravoxel turbulence parameters from ICOSA6 sequence

Number of encoding	Velocity component	Intravoxel standard deviation
1	$V_1 = \cos\theta \cdot u + \sin\theta \cdot v$	$\sigma_1^2 = \cos^2\theta \cdot (\sigma_x^2) + \sin^2\theta \cdot (\sigma_y^2) + 2 \cdot (\cos\theta) \cdot (\sin\theta) \cdot (<u'v'>)$
2	$V_2 = \cos\theta \cdot u - \sin\theta \cdot v$	$\sigma_2^2 = \cos^2\theta \cdot (\sigma_x^2) + \sin^2\theta \cdot (\sigma_y^2) - 2 \cdot (\cos\theta) \cdot (\sin\theta) \cdot (<u'v'>)$
3	$V_3 = \cos\theta \cdot v + \sin\theta \cdot w$	$\sigma_3^2 = \cos^2\theta \cdot (\sigma_y^2) + \sin^2\theta \cdot (\sigma_z^2) + 2 \cdot (\cos\theta) \cdot (\sin\theta) \cdot (<v'w'>)$
4	$V_4 = \cos\theta \cdot v - \sin\theta \cdot w$	$\sigma_4^2 = \cos^2\theta \cdot (\sigma_y^2) + \sin^2\theta \cdot (\sigma_z^2) - 2 \cdot (\cos\theta) \cdot (\sin\theta) \cdot (<v'w'>)$
5	$V_5 = \sin\theta \cdot u + \cos\theta \cdot w$	$\sigma_5^2 = \sin^2\theta \cdot (\sigma_x^2) + \cos^2\theta \cdot (\sigma_z^2) + 2 \cdot (\cos\theta) \cdot (\sin\theta) \cdot (<u'w'>)$
6	$V_6 = \sin\theta \cdot u - \cos\theta \cdot w$	$\sigma_6^2 = \sin^2\theta \cdot (\sigma_x^2) - \cos^2\theta \cdot (\sigma_z^2) + 2 \cdot (\cos\theta) \cdot (\sin\theta) \cdot (<u'w'>)$

* u,v and w indicate the velocity component in three orthogonal directions along x,y, and z.

** <u’v’>, <v’w’> and <u’w’> indicate the Reynolds stress component
